# Origin and location of new Arctic islands and straits due to glacial recession

**DOI:** 10.1007/s13280-018-1041-z

**Published:** 2018-03-29

**Authors:** Wiesław Ziaja, Krzysztof Ostafin

**Affiliations:** Institute of Geography and Spatial Management, Jagiellonian University, Gronostajowa 7, 30-387 Krakow, Poland

**Keywords:** Climate change, Glacial recession, Nearest future scenarios, New Arctic islands and straits

## Abstract

A total of 34 new islands (each 0.5 km^2^ or above) have appeared due to recession of Arctic glaciers under climate warming since the 1960s. Analysis of maps and satellite images of the Arctic coasts has been a basic method of recognizing these islands. Their origin is the final stage of a process which began in the twentieth century. They appear only on the coasts where bedrock elevations above sea level are surrounded by depressions below this level, filled (at least from the landside) with glaciers. Their recession leads to flooding of the depressions by sea water, thus creating straits which separate the new islands from the mainland. Hence, such new islands appear only in Greenland and the European Arctic. Their ecosystems accommodate to new environmental conditions. In the near future, this process will be intensified in a situation of further warming.

## Introduction

Ablation and recession of Arctic glaciers, which expanded during the Little Ice Age and remained advanced until the end of the nineteenth century, is an obvious and widely described effect of the Arctic climate warming since the beginning of the twentieth century. The warming has been interrupted by two minor and short cool periods (with lowering of mean annual temperatures by ca. 1−2 °C) in the 1940s and 1960s (Brázdil 1988; Førland et al. 1997; Kohler et al. 2002). Consequently, the origin of new marine straits is an effect of the glacial recession. These new straits appear in the places where longitudinal depressions in bedrock, situated below sea level and open from both sides, are being abandoned by the lowest parts of the glaciers which previously filled them and being inundated by sea water. In this way, former peninsulas and headlands or other rocky parts of coasts (not only protruding into the sea) are being isolated from a mainland and transformed into new islands. Prior to becoming islands, these features (of bedrock topography) act as pinning points, stabilizing a glacier’s terminus. It is obvious that such islands do not appear due to epeirogenic (i.e., land-forming) processes; on the contrary, the reason for their origin is a progressive decrease in a glacier surface (because the glaciers lying formerly on bedrock undoubtedly belonged to land areas). We do not refer to shelf glaciers because they lie on sea water (and, besides, they are very rare in the Arctic, and no Arctic island has been surrounded by them).

Recession of thick glaciers during the twentieth century was slow but progressive, resulting in a significant decrease in the glaciers’ mass by the 1980s (Zemp et al. 2015). Since the 1980s, a great acceleration of the recession has occurred due to the current large climate warming (Walsh et al. 2011; Styszyńska 2013), and the aforementioned decrease in the glaciers’ mass meant that a decline of their lower parts was much easier. Thus, the first new islands appeared in the 1960s–1990s, and the next ones in increasing number in the twenty-first century.

The objective of this paper is to precisely document the location of new islands which have appeared off the Arctic coasts due to glacier recession under the influence of the current climate warming, and especially to show the effective process of their formation. This objective also refers to the peninsulas and headlands which may be transformed into islands in the near future, because the process of their transformation is both very visible and very advanced. It should be underlined that we do not consider land areas smaller than 0.5 km^2^ (i.e., 50 ha) as islands. Hence, we omit those new smallest Arctic islands which have originated due to the surrounding of small rocky or moraine hills or ridges by the sea, e.g., Fallknatten or Morenetangen near the Vasilievbreen glacier in SE Spitsbergen, Svalbard, illustrated by Pelto (2017), and two islands near the Roze Glacier, Novaya Zemlya (Hydrologists of the Northern Navy 2016), and also due to marine sedimentation in newly inundated marginal zones of glaciers or in shallows, e.g., Yaya in the Laptev Sea (Arctic Satellite Image 2014), etc.

## Materials and methods

Materials for the analysis of the landscape transformations leading to the origin of new islands were mostly collected through a survey of available maps and satellite images of all the Arctic glacial coasts, and especially of the most dynamic sequences of the Arctic icy coastlines, from different times since the 1970s when a satellite survey of the Earth surface began.

The following collections of digital maps at the 1:100 000–1:500 000 scales were examined:

- Danish maps of Greenland (NunaGIS 2017)
- Norwegian maps of Svalbard (TopoSvalbard 2017)
- Russian maps of the Russian Arctic (Soviet military topographic maps 1:200 000 2017)
- Canadian maps of the Canadian Arctic (GeoGratis 2017)
- Landsat from NASA (USGS Global Visualization Viewer 2017) for 1973–2016: Landsat 1–3 MSS, Landsat 4–5 MSS, Landsat 4–5 TM, Landsat 7 SLC, Landsat 8 OLI
- Terra ASTER (ASTER 2017) for 2000–2016
- Sentinel-2 from ESA (Copernicus Open Access Hub 2017) for 2015 and 2016.

**Fig. 1:**
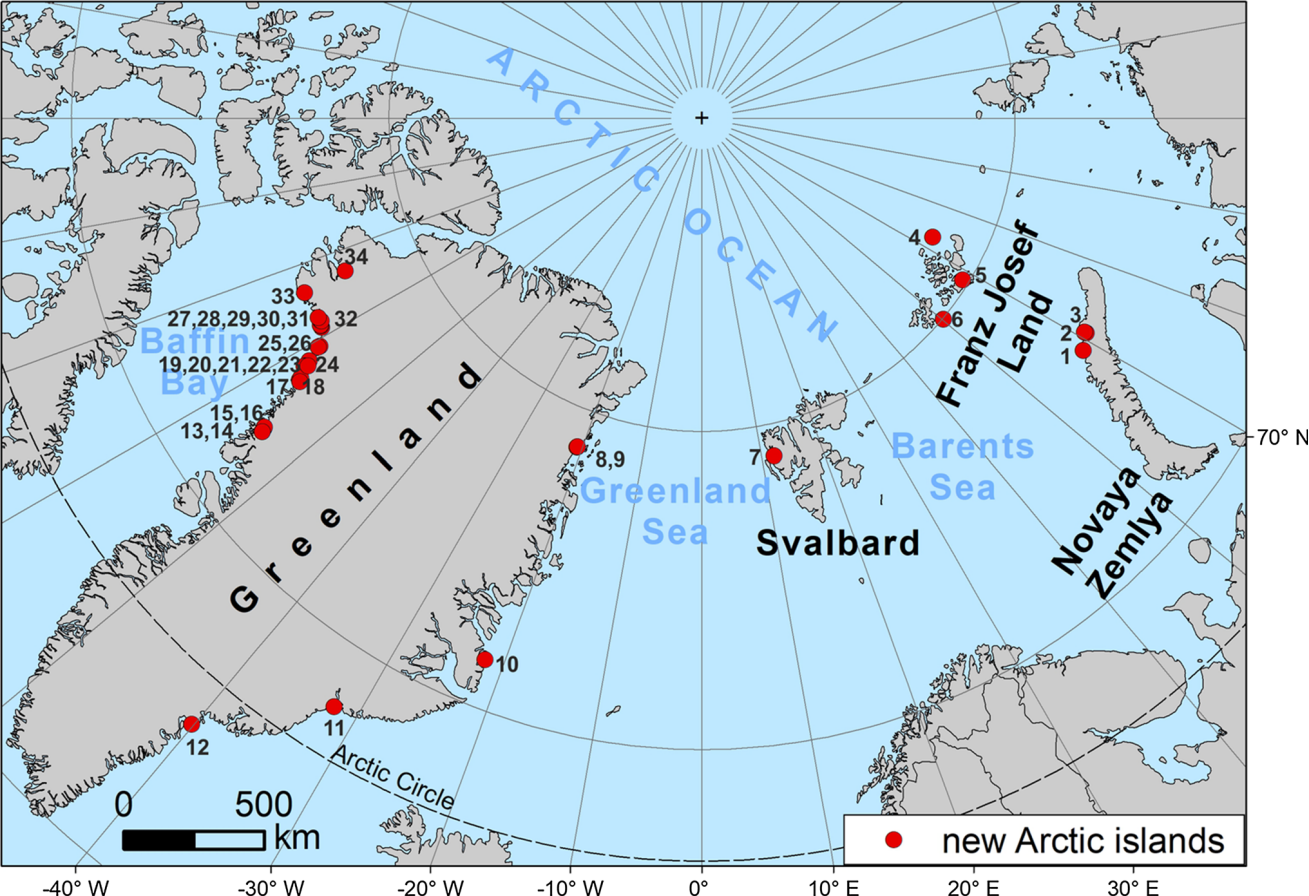
Locations of the new Arctic islands; location numbers are the same as in Table 1

Approximately, an area of 1 560 000 km^2^ of Arctic coasts (including the continental coastlines) has been scanned for new islands by comparing the older maps and satellite images with the newer and newest ones. There are no new islands on the continental coasts of Eurasia and North America. The total of 34 new islands have been discovered in this way (Fig. 1; Table 1), and their origin has been evidenced in the form of a figure containing at least two cartographic pictures—one before the origin of the island (a map or a satellite image) and second after its appearance (all from satellite images). The chosen examples of these figures, which evidence the origin of the new islands, are included in this paper.

**Table 1:** New Arctic islands; location numbers are the same as in Fig. 1

No. location	Name (new or old)	Area (km^2^)	Centroid	Altitude (m)	Year or period of origin
1. Novaya Zemlya	Upor	6.5	75°46′03″N, 58°40′29″E	242	1995–1996
2. Novaya Zemlya	–	0.6	75°57′56″N, 60°20′12″E	< 100	2014
3. Novaya Zemlya	–	0.9	76°01′48″N, 60°48′08″E	< 100	2004
4. Franz Josef Land	Eva-Liv	0.5	81°42′04″N, 62°43′40″E	20	After 1965
5. Franz Josef Land	Hall	59	80°11′30″N, 58°12′9″E	420	2016
6. Franz Josef Land	Northbrook	18	79°57′50″N, 50°11′57″E	308	1985–2006
7. Svalbard	Blomstrandhalvøya	16.3	78°58′47″N, 12°04′50″E	385	1989–1995
8. E Greenland	–	1.7	78°46′54″N, 20°45′36″W	< 100	2013–2015
9. E Greenland	–	3.6	78°46′13″N, 20°50′59″W	< 100	2013–2015
10. E Greenland	Warming	18.7	71°29′12″N, 21°51′23″W	520	2004
11. E Greenland	Kap Deichmann	3.7	68°03′06″N, 32°02′58″W	830	2016–2017
12. E Greenland	Sipulik	4.7	65°01′48″N, 40°07′12″W	100	Before 1972
13. W Greenland	Umanaq	0.7	72°49′34″N, 54°32′44″W	100	1963–1976
14. W Greenland	Umanaq	6.1	72°50′24″N, 54°34′03″W	290	1963–1976
15. W Greenland	Nunnatarsuaq	8.2	72°56′20″N, 54°42′20″W	190	1976–1985
16. W Greenland	Naujavigssuaq	2.8	72°58′22″N, 54°47′01″W	100	1963–1976
17. W Greenland	Nunatakavsak	7	74°41′38″N, 56°45′25″W	280	Before 1985
18. W Greenland	Hovgård Kystland	30.9	74°42′57″N, 56°58′22″W	323	Before 1985
19. W Greenland	Kiataussaptimilia	3.7	74°46′53″N, 57°07′23″W	180	1984–1985
20. W Greenland	Red Head	6.6	75°05′53″N, 58°02′09″W	263	Before 1985
21. W Greenland	–	1.9	75°04′44″N, 58°03′41″W	< 100	2005
22. W Greenland	–	5.6	75°03′57″N, 58°05′25″W	150	2005
23. W Greenland	–	4.2	75°10′28″N, 57°52′21″W	130	2017
24. W Greenland	Tugtuligssûp	17.4	75°18′12″N, 58°18′54″W	370	2014
25. W Greenland	–	6.5	75°46′07″N, 59°11′48″W	220	2003
26. W Greenland	Ivnârqigsorssuaq	2.7	75°49′17″N, 59°12′46″W	400	2014
27. W Greenland	–	0.8	76°10′03″N, 61°13′04″W	220	Before 1973
28. W Greenland	–	3.4	76°10′00″N, 61°27′28″W	230	1983–1988
29. W Greenland	–	1.3	76°10′01″N, 61°31′25″W	100	1989–1990
30. W Greenland	–	0.6	76°15′10″N, 61°59′38″W	< 100	2009–2010
31. W Greenland	–	6.6	76°13′57″N, 62°05′45″W	310	1988
32. W Greenland	–	8.8	76°13′15″N, 62°34′55″W	415	Before 1972
33. W Greenland	–	1.5	76°10′08″N, 66°18′17″W	< 100	2006–2007
34. W Greenland	Josephine Peary Ø	16.8	77°36′59″N, 66°50′33″W	500	1982–1987

The second method of collecting data was a survey of the literature on new islands, mainly on the Internet. This literature consists mostly of three kinds of popularized scientific notes: (1) originally observed, denoted, and published, as indicated by Pelto (2009–2017), plus many compiled by Pelto (2017) on different Arctic coasts, and by Sharov (2014) on the Eurasian Arctic coasts, (2) notes in Wikipedia (Island of Yurij Kuchiev 2013), and other Internet pages without authors’ names (World Climate Reports 2008), and (3) news from press agencies (e.g., RIANOVOSTI 2012). As a rule, the appearance of the new Arctic islands due to glacial recession is not evidenced in the international scientific journals, apart from rare exceptions (e.g., Ziaja and Ostafin 2015). However, papers on the entire recession of the tide-water glaciers are being published in these journals, and some of these papers may help in determining the course and circumstances of the coastal landscape transformation. These papers (Box and Decker 2011; Howat and Eddy 2011) directed our attention to some segments of the Greenland coast where we found new islands in the satellite data.

Direct summer field investigations of landscape changes under glacial recession, including landscape mapping, which referred to this problem were carried out by us in Spitsbergen, the largest island of Svalbard, namely on the new island in Kongsfjorden in 1995 and on the glacial isthmus being transformed into a new sound between the Sørkapp Land peninsula and the rest of Spitsbergen. We explored the isthmus between the Hornbreen and Hambergbreen tide-water glaciers in both August 2005 (Ziaja and Ostafin 2015) and August 2016, identifying its rapid thinning and recession. The ice divide between two isthmus’ glaciers was lowered by 20 m (from 180 to 160 m at the pass) during this 11-year period. The glaciers’ thickness below was lessened by a similar value which resulted in the decrease of the isthmus’ width from 7.9 km in 2005 to 5.6 km in 2016. Such a quick recession of glaciers was observed by us over the whole of the Sørkapp Land peninsula during the twenty-first century (Ziaja et al. 2009; Ziaja and Dudek 2016). The recession is really impressive on Isbukta where a few islets (smaller than 0.5 km^2^) appeared or are developing due to the retreat of the Vasilievbreen and Skilfonna glaciers (Pelto 2017).

## Results

The largest number of islands have originated due to the recession (lowering and shortening) of a single glacial tongue which formed one coast of a peninsula or headland having a form of a rocky elevation situated at the end of this tongue.

Some glaciers formed the coast of a peninsula or headland not surrounding them in the Little Ice Age (though then being larger, thicker and wider). A majority of the new Greenland islands, the new island in Spitsbergen (Svalbard), and two new islands in Novaya Zemlya originated due to the recession of such glaciers.

**Fig. 2:**
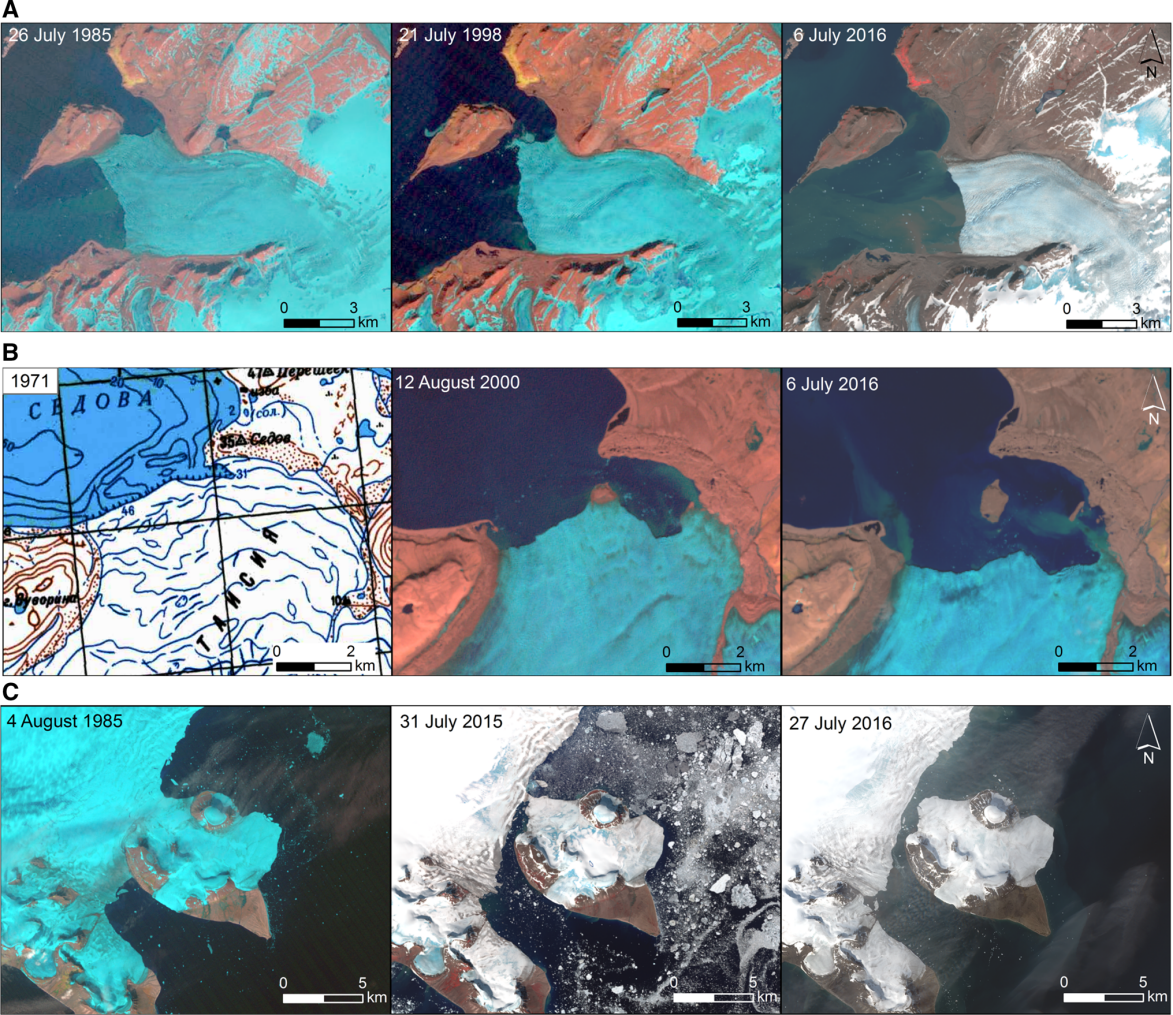
Transformation of coastal areas into new islands, Novaya Zemlya and Franz Josef Land: **a** transformation of the Upor Headland into the new island (No. 1 in Table 1; Fig. 1) due to the recession of the Krivosheina Glacier at the Novaya Zemlya coast, **b** the new island’s (No. 2 in Table 1; Fig. 1) appearance from under the Taisiya Glacier at the Novaya Zemlya coast, **c** transformation of the SE part (Littrov Peninsula) of the Hall Island (No. 5 in Table 1; Fig. 1), Franz Josef Land, into the new island due to the recession of two glaciers. Map excerpt from Soviet military topographic map (2017)

Figure 2a shows the example from Novaya Zemlya where the Upor Headland was transformed into a new island due to the recession of the glacier which had previously reached its SE coast. This also refers to the small unnamed new island at the front of the Chernysheva Glacier (No. 3 in Table 1). The last two examples are also described by Pelto (2017).

**Fig. 3:**
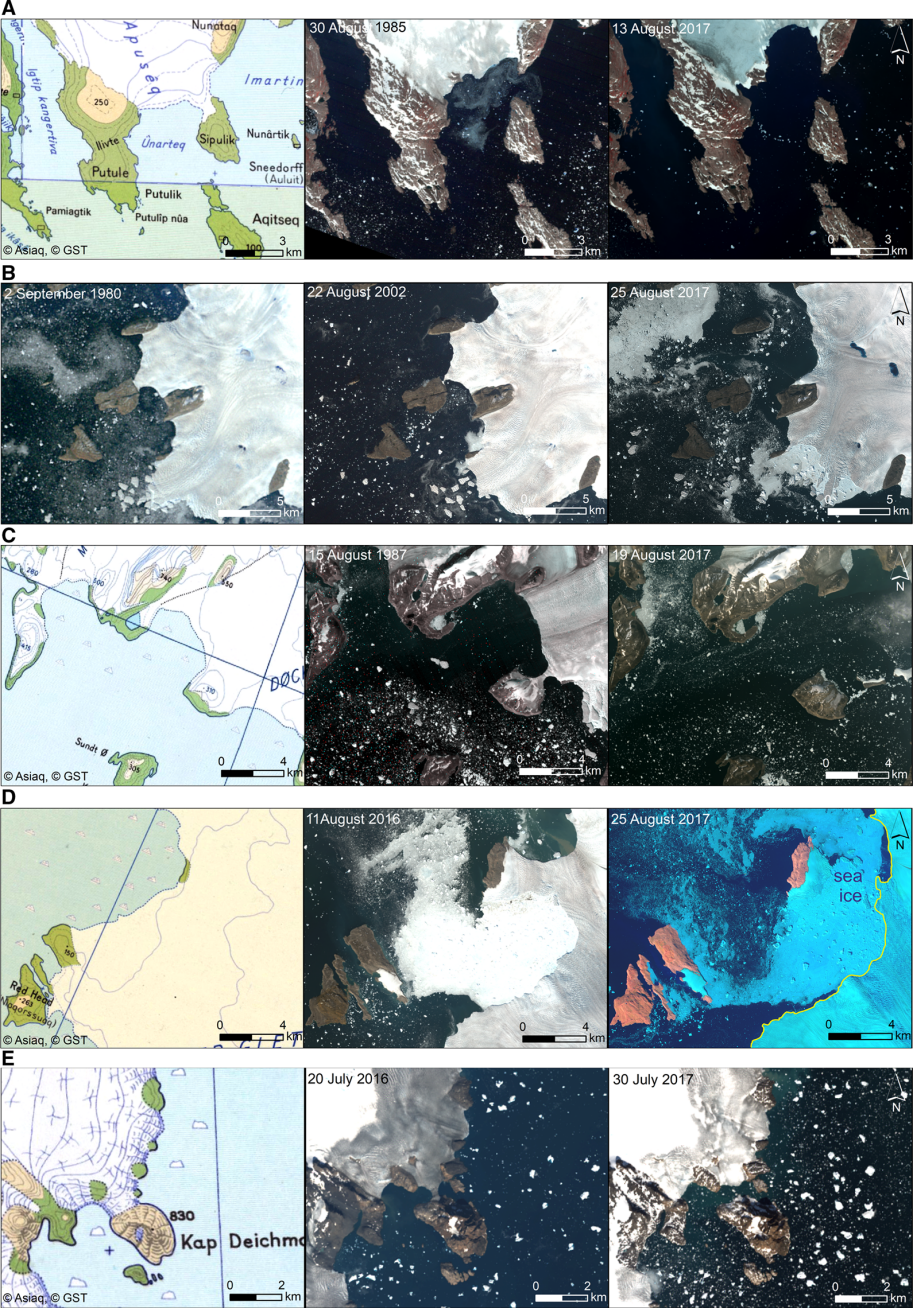
Transformation of coastal areas into new islands, Greenland: **a** transformation of the Sipulik peninsula into a new island (No. 12 in Table 1; Fig. 1) due to the recession of the Apusêq Glacier on the SE Greenland coast, probably another island is appearing from under the glacier; **b** transformation of the unnamed peninsula and Ivnârqigsorssuaq headland (in the north) into new islands (Nos. 25 and 26 in Table 1; Fig. 1) due to the recession of the Nordenskiöld Glacier on the NW Greenland coast (the Nansen Glacier is visible to the south); **c** transformation of an unnamed peninsula and headland (Nos. 31 and 32 in Table 1; Fig. 1) into new islands due to the recession of the Morell and Døcker Smith Glaciers on the NW Greenland coast; **d** transformation of the Red Head peninsula and three unnamed coastal areas into new islands (Nos. 20–23 in Table 1; Fig. 1) due to the recession of the Kjer and Steenstrup Glaciers (the latter one to the north) on the NW Greenland coast; **e** transformation of Cape Deichmann into a new island (No. 11 in Table 1; Fig. 1) due to the recession of the Hutchinson Glacier on the SE Greenland coast. Original information of the maps used here: topographic map 1:500,000 and 1:250,000 displayed at different zoom levels. The map is a mosaic of scanned paper maps produced since the 1940s. Copyright Geodata Agency (NunaGIS 2017)

In SE Greenland, the former Sipulik peninsula (Fig. 3a) and Cape Deichmann (Fig. 3e) were separated from the mainland in a very similar way, due to the declining of the glacier tongues reaching both the peninsula and the cape from the north. Sipulik became an island before 1985 but Cape Deichmann is a very new island, formed in 2016 or 2017. The largest number of new islands have appeared on the NW Greenland coast due to the recession of large outlet glaciers of the Greenland Ice Sheet since the 1980s. For detailed descriptions of widespread glacier retreat on the coasts of NW Greenland, see Pelto (2009–2017). Figure 3b shows the origin of two new islands due to the recession of the Nordenskiöld Glacier, Fig. 3c shows the appearance of the next two islands due to the recession of the Morell and Døcker Smith Glaciers. The retreat of the Melville Bay icy coastline from 1916 to 2009 was described by Van As (2011). Four new islands were formed as a result of the recession of the Kjer and Steenstrup Glaciers (Fig. 3d).

However, some glaciers are so extensive that they have surrounded rocky elevations and in rare cases covered them, and only after a protracted period of recession, i.e., in the second half or at the end of the twentieth century, they have been in contact on only one side of a peninsula or headland, leading to the formation of a strait or island. At least one new Greenland island, shown by Van As (2011) as a nunatak until 2009 and as the northernmost island in Fig. 3d, and one island at the front of the Taisiya Glacier in Novaya Zemlya (Fig. 2b), appeared in this way, as is also shown by Pelto (2017). In addition, two new islets were uncovered from under the Vilkitskogo and Nizkiy glaciers during their recession between 1990 and 2015 (Pelto 2017).

A smaller number of straits or sounds (and islands) have originated due to the recession of two or more glaciers which previously grew from two sides. All the three new islands in Franz Josef Land, which were separated from the Eva-Liv, Hall, and Northbrook islands (Nos. 4, 5, and 6 in Table 1; Sharov 2014), including the youngest island (Fig. 2c), and Warming Island in Greenland, which is the best known new island (World Climate Report 2008), appeared due to the recession of at least two glaciers which had previously been confluent.

## Islands in the course of forming

Current observations of the progressive decline of some tide-water glaciers connecting peninsulas with a mainland evidence their quick transformation into new straits (sounds), which leads to the origin of new islands.

**Fig. 4:**
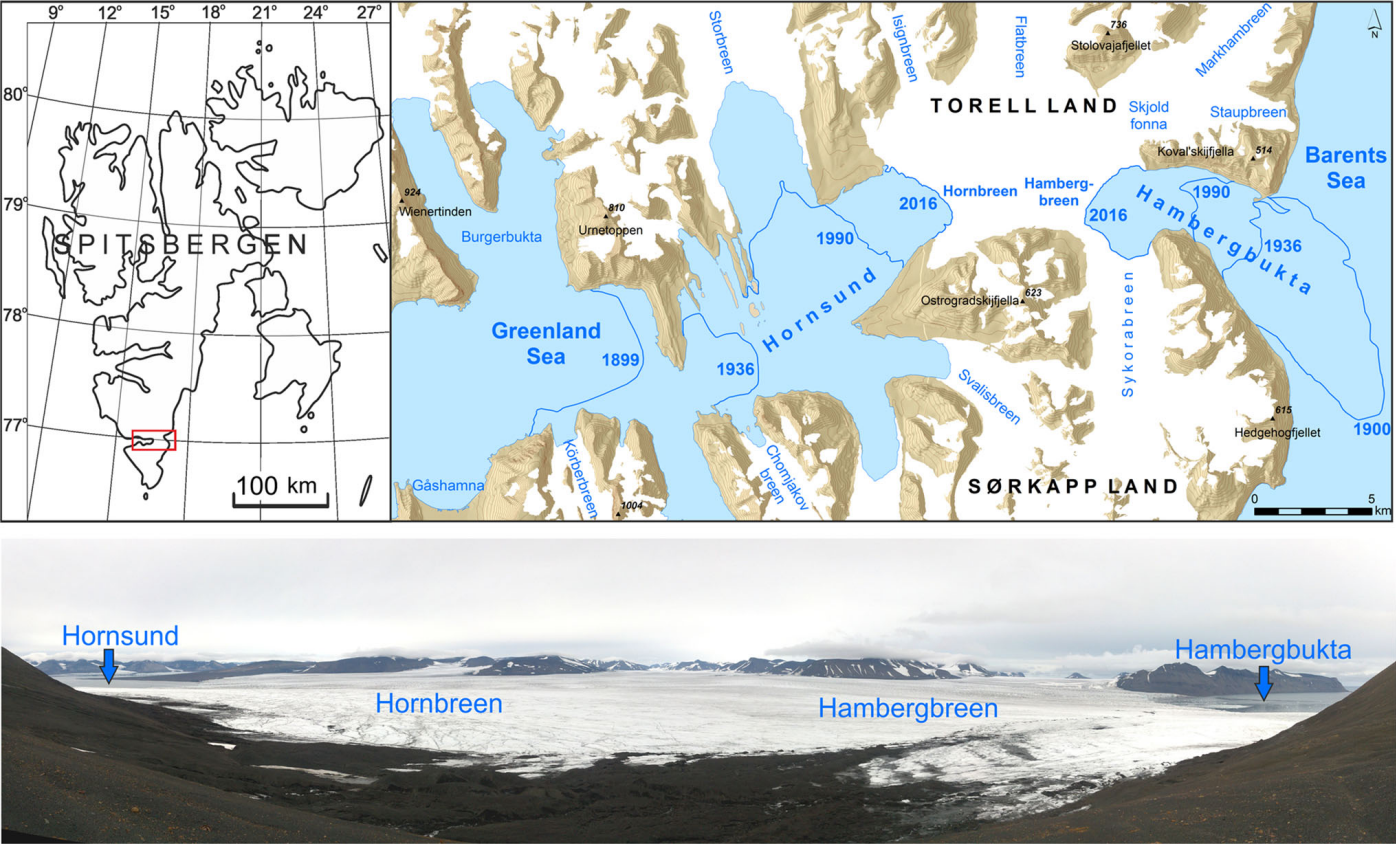
Progressive narrowing of the isthmus (built of two glaciers) connecting the Sørkapp Land peninsula with the rest of Spitsbergen (from above 30 km in 1899 to 5.6 km in 2016) can result in transforming Sørkapp Land into a new island (*upper left-hand map* location,* upper right-hand map* detail). Extent of tide-water glaciers (years shown on *upper right-hand map*): in 1899–1900 according to Wassiliew (1925), in 1936 according to the old Norwegian topographical map (C12 Markhambreen 1956; C13 Sørkapp 1986), in 1990, according to the new Norwegian topographical map (C12 Markhambreen 2008; C13 Sørkapp 2007); general topography is also taken from the same maps (C12 Markhambreen 2008; C13 Sørkapp 2007); and extent of glaciers in 2016 according to Sentinel-2 satellite images (Copernicus Open Access Hub 2017). *Lower image* looking north: the glacial isthmus situated between two fjords, Hornsund from the west and Hambergbukta from the east, in summer 2016; photo, K. Ostafin

A key example of a potential new island in the process of forming is that of Sørkapp Land, the southernmost Spitsbergen peninsula (Ziaja and Ostafin 2005; Sharov and Zaprudnova 2014; Ziaja and Ostafin 2015; Grabiec et al. 2017). It is located south of the heads of the Hornsund and Hambergbukta fjords (Fig. 4), where the recession of the Hornbreen and Hambergbreen glaciers will lead to a new island forming. Pälli et al. (2003) noted that Hornbreen retreated by 13.5 km and Hambergbreen by 16 km from 1899–1900 to 2000. Pälli et al. (2003) found that there is no below-sea-level connection underneath the Hornbreen–Hambergbreen divide which would separate Sørkapp Land from Torrell Land. The ice divide of Hornbreen–Hambergbreen is below the local snowline at 300 m, and Pälli et al. (2003) indicated that it cannot survive the current climate change. In 1983, the distance from the terminus of Hornbreen to the terminus of Hambergbreen was 17 km, while, in 2013, it was 9 km (Pelto 2017). However, the newest and more advanced radar sounding of the declining glacier isthmus indicates that the ice is grounded well below sea level. The result will be a strait at least ca. 40 m deep (Grabiec et al. 2017).

Two new islands may appear in Novaya Zemlya, at the front of the Krayniy Glacier with continued recession as illustrated by Pelto (2017). Also, the same process of progressive narrowing of the glacial connection between the former Littrov Peninsula and the rest of the Hall Island, Franz Josef Land, continued until 2015, resulting in island formation in 2016 (Fig. 2c). This was analyzed by Sharov and Zaprudnova (2014).

It is noteworthy that an unnamed large peninsula (ca. 200 km^2^) is close to separation from the mainland on the eastern Greenland coast (76.5°N) at Dove Bay. The new island and strait will be probably formed in 2018–2019, after the decline of the glacier tongue which connects the peninsula with the mainland.

## Recapitulation and Discussion

Climate warming is a proximal cause of the appearance of new islands. A direct reason for the origin of the new islands is sea transgression into depressions abandoned by glacial recession due to increased ablation and/or calving. This process leads to the fragmentation of the heavily glaciated coasts and formation of new straits.

The rate of island formation has been increasing: six new islands appeared prior to 1980, 12 were formed in the period 1980–2000, and 15 since 2000. One island, the smallest one (0.5 km^2^), appeared after 1965 but at an unknown date (Table 1).

Sea transgression (i.e., the origin of new straits in the lowest parts of tide-water glaciers) did not result in a rise of sea level. However, the negative net mass balance of the glaciers (being the result of climate warming and the cause of the glacier recession), necessary for all the described processes, contributes to this rise.

It is necessary to distinguish the origin of new islands from the release of the existing (old) islands from flat and land-fastened sea ice. This ice was very stabile and multiannual until the 1980s, and has since begun to decline in places due to an intensified climate warming. Such a release leads to a complete environmental transformation of the existing islands but does not create new ones. Hence, we do not refer to this process.

Some authors (e.g., Sharov 2006; Zaprudnova 2014) use the term “ice bridge” for a glacier or glaciers filling up a potential strait. This is an inaccurate description because glacier ice is not floating on sea water in the cases reviewed here.

**Fig. 5:**
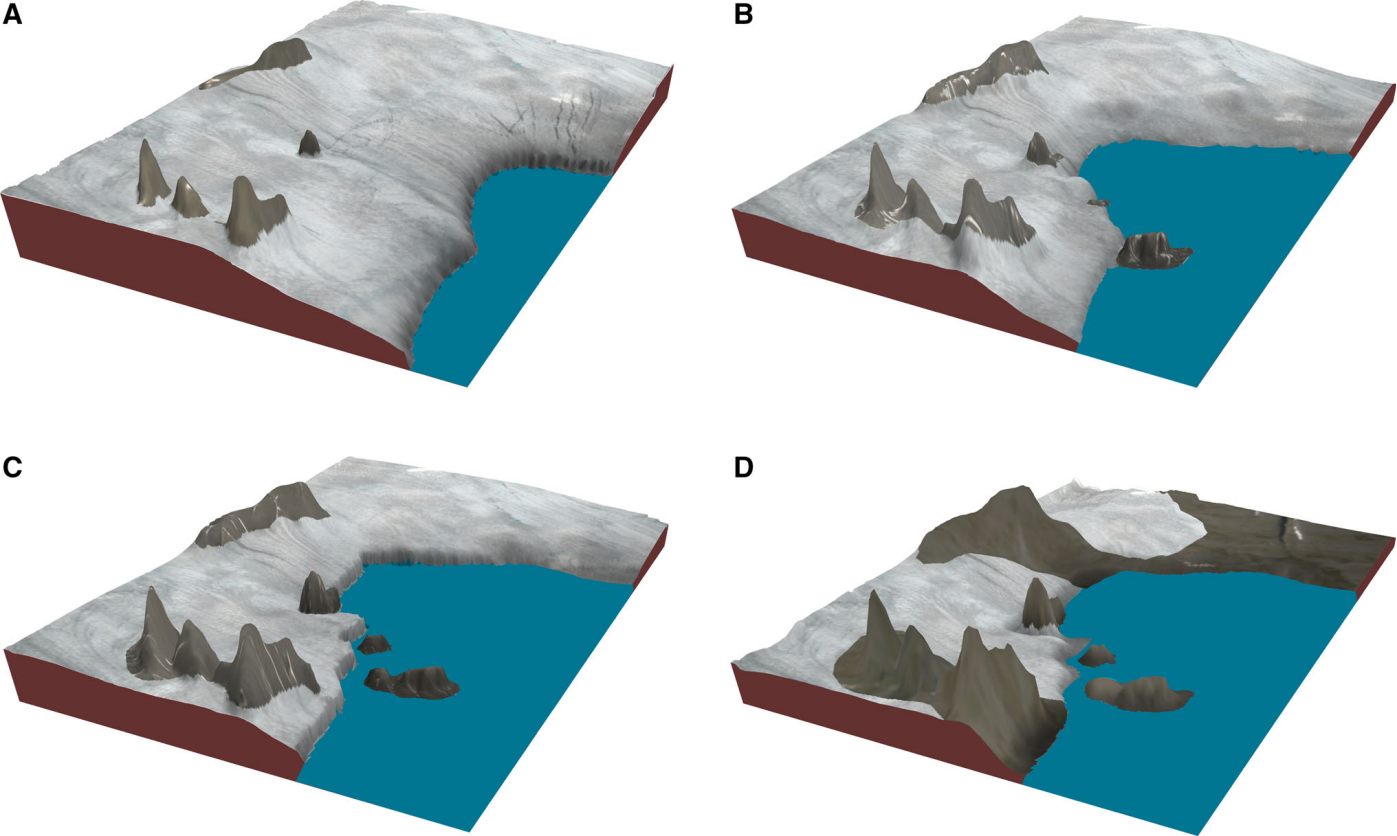
Simplified model of coastal landscape transformation under the influence of climate warming and glacial recession leading to the appearance of the new islands: **a** first stage just after the Little Ice Age (at the beginning or middle of the twentieth century), **b** second stage (at the middle or end of the twentiethth century), **c** third stage (at the end of the twentiethth century or the beginning of the twenty-first century), **d** fourth stage (at the beginning of the twenty-first century)

The methods of new islands’ formation described above may be connected into one cycle of coastal landscape development under the influence of climate warming and glacial recession, which is shown in the model illustrated in Fig. 5.

In its first stage, all the coast is covered with thick glaciers which form an icy coastline (Fig. 5a). There may be nunataks (peaks or hills surrounded by glaciers) in the inland off this coastline. In the second stage, after a few or several dozen years of climate warming, the glaciers are shrinking and a new rocky coast, with headlands and peninsulas (some of them are being transformed from former nunataks), is becoming partly ice-free. These peninsulas and headlands are firmly connected to their adjacent mainland by glaciers, and these glacial connections of the headlands and peninsulas with a mainland become smaller and more limited at this stage as coastal change progresses (Fig. 5b). In the third stage, the glacial connections disappear or are being disrupted. If they filled rocky depressions below the sea level, these depressions are being inundated by sea water and changed to transformed straits separating the new islands from their mainland (Fig. 5c). Finally, in the fourth stage, a major part of a coast becomes free of ice and the glaciers split due to a huge decrease in their thickness. A new unglaciated landscape develops near the much reduced glaciers (Fig. 5d). Some glacial coastlines covered by the maximum extents of glaciers might miss out the first stage, and many of them (especially in the northern parts of the Arctic archipelagos) did not reach the fourth stage.

Intensive climate warming, which is predicted by IPCC (2014), will accelerate this relatively quick current recession of Arctic glaciers and isolate additional islands.

## Conclusions

The new straits have already shortened some sea routes along the Greenland coast, and some Arctic sea-ways will shorten with continued climate warming. A new strait across today’s southern Spitsbergen (Svalbard)—which would appear due to the connection of the Hornsund and Hambergbukta fjords—would be of a great importance from both the politico-economical and environmental (ecological) points of view.

The new islands are not economically important. However, their ecosystems must accommodate to new environmental conditions. Replacing the glacial ice with sea water is surely favorable for life expansion on them.

The new islands and straits appear only in predisposed parts of the Arctic coasts where bedrock elevations above sea level are surrounded by depressions below sea level, filled (at least from the landside) with glaciers. Recession of these glaciers leads to the flooding of the depressions by sea water and thus the creation of new straits separating the former parts of coasts from a mainland.

Hence, there are many new islands that have formed and are forming in Greenland, and a few new islands in the European Arctic (Novaya Zemlya, Franz Josef Land, Svalbard), but fewer (practically none at the moment) in the Canadian, American, and Siberian Arctic, in spite of the progressive recession of glaciers there (it is not unlikely in future, although we do not know the bedrock topography under contemporary glaciers).

In the nearest future, the described process of origin of new islands will continue in the case of the stabilizing of the current climate conditions, or be intensified in the case of a further climate warming as anticipated by the IPCC (2014).
